# Supplementary material for: Geographical barriers and multimorbidity in quilombola territories of the amazon region
Source: PLoS One. 2026 Mar 27;21(3):e0344043. doi: 10.1371/journal.pone.0344043 (PMC13028379; doi:10.1371/journal.pone.0344043)
Supplement: Attachment — Submitted filename: PONE-D-25-65345_review.pdf [file pone.0344043.s001.pdf]

PLOS One

Geographical barriers and multimorbidity in quilombola territories of the amazon region

--Manuscript Draft--

**Manuscript Number:**	PONE-D-25-65345
**Article Type:**	Research Article
**Full Title:**	Geographical barriers and multimorbidity in quilombola territories of the amazon region
**Short Title:**	Geographical barriers and multimorbidity in quilombola territories
**Corresponding Author:**	Livia de Aguiar Valentim<br>University of Pará State Centre for Biological and Health Sciences: Universidade do Estado do Para Centro de Ciencias Biologicas e da Saude<br>Santarem, BRAZIL
**Keywords:**	Quilombola Communities; Social Determinants of Health, Health Services Accessibility; Multimorbidity
**Abstract:**	Background<br><br>Quilombola communities in the Brazilian Amazon face persistent social and territorial inequities that shape health outcomes and access to care. Geographic isolation, limited transportation, centralization of specialized services, and socioeconomic disadvantages contribute to unequal opportunities for timely diagnosis and treatment. Understanding how these determinants interact with patterns of multimorbidity is essential for guiding equiTable health policies and strengthening primary care in remote territories.<br><br>Methods<br><br>A cross-sectional epidemiological study was conducted with 518 adults from nine quilombola communities in Santarém, Pará. Data were collected through household surveys addressing sociodemographics, self-reported diseases, service utilization and resolvability. Geographic coordinates of communities and health services were mapped to classify accessibility as high, medium or low. Diseases were converted into a binary matrix to estimate prevalence and identify multimorbidity (≥2 conditions). Statistical analyses included chi-square tests, ANOVA, Spearman correlations and heatmap visualization. A Composite Access Index (CAI) integrating geographic distance, epidemiological burden and service-use indicators was developed. A Random Forest model was used to identify conditions most strongly associated with multimorbidity.<br><br>Results<br><br>Communities showed marked territorial heterogeneity. Pérola do Maicá had the highest accessibility, while Ituqui, Tiningu and Murumuru presented substantial geographic and logistical barriers. Service utilization ranged from 42.9% to 95.0%, and most communities relied on care outside their territory (70–95%). Complete problem resolution was reported by 72.5% of participants, though with variation among communities. The CAI identified Ituqui (0.550), Tiningu (0.480) and Murumurutuba (0.331) as the most vulnerable territories. The Random Forest model achieved 93.6% accuracy, with hypertension, diabetes, musculoskeletal diseases, arthritis/rheumatism and heart disease emerging as key predictors of multimorbidity.<br><br>Discussion<br><br>Findings reveal that social and territorial determinants operate synergistically, reinforcing inequities in access, continuity of care and disease burden.<br><br>Conclusions<br><br>Geographical barriers and service distribution shape multimorbidity patterns and access among quilombola populations. Strengthening primary care, transportation and

	diagnostic support is essential to reduce inequities and improve health outcomes in remote Amazonian territories.
**Order of Authors:**	Leanna Silva Aquino
	Ellen Mara Fernandes da Silva
	Victoria Valentim Aguiar
	Cesar Ferreira Fernandes Filho
	Sheyla Mara Silva de Oliveira
	Tatiane Costa Quaresma
	Valney Mara Gomes Conde
	Nádia Vicência do Nascimento Martins
	Marcos Manoel Honorato
	Veridiana Barreto do Nascimento
	Guilherme Augusto Barros Conde
	Franciane de Paula Fernandes
	Livia de Aguiar Valentim
**Additional Information:**	
**Question**	**Response**
**Financial Disclosure**<br><br>Enter a financial disclosure statement that describes the sources of funding for the work included in this submission. Review the submission guidelines for detailed requirements. View published research articles from *PLOS ONE* for specific examples.<br><br>This statement is required for submission and **will appear in the published article** if the submission is accepted. Please make sure it is accurate.<br><br>**Funded studies**<br>Enter a statement with the following details:<br>• Initials of the authors who received each award<br>• Grant numbers awarded to each author<br>• The full name of each funder<br>• URL of each funder website<br>• Did the sponsors or funders play any role in the study design, data collection and analysis, decision to publish, or preparation of the manuscript?	The author(s) received no specific funding for this work.

Powered by Editorial Manager® and ProduXion Manager® from Aries Systems Corporation

Did you receive funding for this work?	
**Competing Interests**<br><br>Use the instructions below to enter a competing interest statement for this submission. On behalf of all authors, disclose any competing interests that could be perceived to bias this work—acknowledging all financial support and any other relevant financial or non-financial competing interests.<br><br>This statement is required for submission and **will appear in the published article** if the submission is accepted. Please make sure it is accurate and that any funding sources listed in your Funding Information later in the submission form are also declared in your Financial Disclosure statement.<br><br>View published research articles from *PLOS ONE* for specific examples.<br><br>**NO authors have competing interests**<br><br>Enter: *The authors have declared that no competing interests exist.*<br><br>**Authors with competing interests**<br><br>Enter competing interest details beginning with this statement:<br><br>*I have read the journal's policy and the authors of this manuscript have the following competing interests: [insert competing interests here]*<br><br>* typeset	The authors have declared that no competing interests exist.
**Ethics Statement**<br><br>Enter an ethics statement for this submission. This statement is required if the study involved:<br><br>• Human participants<br>• Human specimens or tissue	The study followed the ethical standards established by Resolution CNS No. 466/2012 and complied with the principles of the Declaration of Helsinki. Participant recruitment occurred between September 1, 2024, and October 30, 2025. All individuals invited to participate received detailed information about the study objectives and procedures, and written informed consent was obtained prior to data collection. Signed consent forms were securely stored as part of the study documentation, ensuring verification of voluntary participation. The research protocol was approved by the Research Ethics Committee of the Universidade do Estado do Pará (UEPA), under opinion No.

• Vertebrate animals or cephalopods<br>• Vertebrate embryos or tissues<br>• Field research<br><br>Write "N/A" if the submission does not require an ethics statement.<br><br>General guidance is provided below. Consult the submission guidelines for detailed instructions. **Make sure that all information entered here is included in the Methods section of the manuscript.**	4.915.684, guaranteeing confidentiality, autonomy, and the protection of participants' rights throughout all stages of the research.

Powered by Editorial Manager® and ProduXion Manager® from Aries Systems Corporation

**Format for specific study types**<br><br>**Human Subject Research (involving human participants and/or tissue)**<br>• Give the name of the institutional review board or ethics committee that approved the study<br>• Include the approval number and/or a statement indicating approval of this research<br>• Indicate the form of consent obtained (written/oral) or the reason that consent was not obtained (e.g. the data were analyzed anonymously)<br><br>**Animal Research (involving vertebrate animals, embryos or tissues)**<br>• Provide the name of the Institutional Animal Care and Use Committee (IACUC) or other relevant ethics board that reviewed the study protocol, and indicate whether they approved this research or granted a formal waiver of ethical approval<br>• Include an approval number if one was obtained<br>• If the study involved *non-human primates*, add *additional details* about animal welfare and steps taken to ameliorate suffering<br>• If anesthesia, euthanasia, or any kind of animal sacrifice is part of the study, include briefly which substances and/or methods were applied<br><br>**Field Research**<br><br>Include the following details if this study involves the collection of plant, animal, or other materials from a natural setting:<br>• Field permit number<br>• Name of the institution or relevant body that granted permission	
**Data Availability**<br><br>Authors are required to make all data underlying the findings described fully available, without restriction, and from the time of publication. PLOS allows rare exceptions to address legal and ethical concerns. See the PLOS Data Policy and FAQ for detailed information.	No - some restrictions will apply

Powered by Editorial Manager® and ProduXion Manager® from Aries Systems Corporation

A Data Availability Statement describing where the data can be found is required at submission. Your answers to this question constitute the Data Availability Statement and **will be published in the article**, if accepted.<br><br>**Important:** Stating 'data available on request from the author' is not sufficient. If your data are only available upon request, select 'No' for the first question and explain your exceptional situation in the text box.<br><br>Do the authors confirm that all data underlying the findings described in their manuscript are fully available without restriction?	
**Describe where the data may be found in full sentences. If you are copying our sample text, replace any instances of XXX with the appropriate details.**<br><br>• If the data are **held or will be held in a public repository**, include URLs, accession numbers or DOIs. If this information will only be available after acceptance, indicate this by ticking the box below. For example: *All XXX files are available from the XXX database (accession number(s) XXX, XXX.*).<br>• If the data are all contained **within the manuscript and/or Supporting Information files**, enter the following: *All relevant data are within the manuscript and its Supporting Information files.*<br>• If neither of these applies but you are able to provide **details of access elsewhere**, with or without limitations, please do so. For example:<br><br>*Data cannot be shared publicly because of [XXX]. Data are available from the XXX Institutional Data Access / Ethics Committee (contact via XXX) for researchers who meet the criteria for access to confidential data.*<br><br>*The data underlying the results presented in the study are available from (include the name of the third party*	The data analyzed in this study are not publicly available due to privacy and ethical restrictions. The dataset used for this research contains sensitive patient in-formation and is subject to confidentiality agreements. Therefore, the data cannot be shared openly. However, researchers interested in accessing the data can contact the corresponding author and request access, subject to the approval of the ethics committee and compliance with relevant data protection regulations.

and contact information or URL).<br>• This text is appropriate if the data are owned by a third party and authors do not have permission to share the data.<br><br>* typeset	
Additional data availability information:	

Powered by Editorial Manager® and ProduXion Manager® from Aries Systems Corporation

Manuscript
Click here to access/download;Manuscript;artigo leanna 2.docx

Geographical barriers and multimorbidity in quilombola territories of the amazon region

Leanna Silva Aquino[1&], Ellen Mara Fernandes da Silva[1&], Victoria Valentim Aguiar[4&], Cesar Ferreira Fernandes Filho[5&], Sheyla Mara Silva de Oliveira[2&], Tatiane Costa Quaresma[2&], Valney Mara Gomes Conde[2&], Nádia Vicência do Nascimento Martins[2&], Marcos Manoel Honorato[2&], Veridiana Barreto do Nascimento[2&], Guilherme Augusto Barros Conde[6&], Franciane de Paula Fernandes[2&], Lívia de Aguiar Valentim[3&*]

[1]Academic of the State University of Pará – UEPA, Santarém, Pará, Brasil

[2]Assistant professor State University of Pará – UEPA, Santarém, Pará, Brasil

[3]PhD in Sciences at the University of São Paulo (USP), Assistant Professor of the Nursing Course at the University of the State of Pará, Santarém, Pará, Brasil

[4]Academic of the Metropolitan College of Pará – FAMETRO, Santarém, Pará, Brasil

[5]Academic of the University of Amazonia – UNAMA, Santarém, Pará, Brasil

[6]Assistant professor Federal University of West Para- UFOPA, Santarém, Pará, Brasil

[3]corresponding author: livia.valentim@uepa.br

[&] These authors also contributed equally to this work

Abstract

Background: Quilombola communities in the Brazilian Amazon face persistent social and territorial inequities that shape health outcomes and access to care. Geographic isolation, limited transportation, centralization of specialized services, and socioeconomic disadvantages contribute to unequal opportunities for timely diagnosis and treatment. Understanding how these determinants interact with patterns of multimorbidity is essential for guiding equiTable health policies and strengthening primary care in remote territories. **Methods:** A cross-sectional epidemiological study was conducted with 518 adults from nine quilombola communities in Santarém, Pará. Data were collected through household surveys addressing sociodemographics, self-reported diseases, service utilization and resolvability. Geographic coordinates of communities and health services were mapped to classify accessibility as high, medium or low. Diseases were converted into a binary matrix to estimate prevalence and identify multimorbidity (≥2 conditions). Statistical analyses included chi-square tests, ANOVA, Spearman correlations and heatmap visualization. A Composite Access Index (CAI) integrating geographic distance, epidemiological burden and service-use indicators was developed. A Random Forest model was used to identify conditions most strongly associated with multimorbidity. **Results:** Communities showed marked territorial

heterogeneity. Pérola do Maicá had the highest accessibility, while Ituqui, Tiningu and Murumuru presented substantial geographic and logistical barriers. Service utilization ranged from 42.9% to 95.0%, and most communities relied on care outside their territory (70–95%). Complete problem resolution was reported by 72.5% of participants, though with variation among communities. The CAI identified Ituqui (0.550), Tiningu (0.480) and Murumurutuba (0.331) as the most vulnerable territories. The Random Forest model achieved 93.6% accuracy, with hypertension, diabetes, musculoskeletal diseases, arthritis/rheumatism and heart disease emerging as key predictors of multimorbidity. **Discussion:** Findings reveal that social and territorial determinants operate synergistically, reinforcing inequities in access, continuity of care and disease burden. **Conclusions:** Geographical barriers and service distribution shape multimorbidity patterns and access among quilombola populations. Strengthening primary care, transportation and diagnostic support is essential to reduce inequities and improve health outcomes in remote Amazonian territories.

Keywords: Quilombola Communities; Social Determinants of Health, Health Services Accessibility; Multimorbidity

Introduction

Quilombola communities in the Amazon region of Pará have experienced, for decades, a set of social inequalities that manifest directly in the health-disease process and in access to health services. These inequalities are rooted in historical, political, and economic conditions that shape the distribution of opportunities across the territory, producing persistent scenarios of vulnerability. The literature shows that factors such as income, education, occupation, environmental conditions and territorial characteristics strongly influence the possibilities of care, positioning the social determinants of health (SDH) as a central framework for understanding inequities among different population groups [1-3].

In the Brazilian context, SDH have been recognized as factors that determine how individuals and communities become ill, remain healthy and access services provided by the Unified Health System (SUS). Models such as Dahlgren and Whitehead’s highlight that structural elements, including public policies, living and working conditions, and socioeconomic factors, interact with individual characteristics and lifestyles to produce avoidable and unjust inequalities [4]. Among traditional populations, such as quilombolas, these layers of determination become even more complex, as geographic

barriers, territorial isolation, limited service availability and structural constraints overlap, reinforcing cycles of illness and reducing the resolvability of care [5-7].

The theory of health inequities proposed by Michael Marmot further deepens this analysis by demonstrating how historically constructed social inequalities reverberate in measurable differences in health outcomes. According to the author, material conditions, socioeconomic position and the social meanings attributed to these conditions shape trajectories of illness and well-being. Recognizing how such inequalities are distributed across territories and among marginalized populations is therefore essential [2,3]. In quilombola communities, this perspective reveals that insufficient access to essential goods and services, such as education, income and sanitation infrastructure represents not only material scarcity but also rights violations with direct impacts on daily life.

Recent studies in quilombola territories have shown that difficulties in transportation, long distances to health units, absence of regular transit, limited access to river-based emergency services and dependence on privately owned boats in emergencies constitute a reality that directly affects the ability to seek timely care [7-9]. These constraints are compounded by socioeconomic restrictions, underdiagnosis and challenges in continuity of care, reinforcing that territory is not merely a backdrop but an active determinant of health.

Understanding the social determinants that influence access to health care in the quilombola communities of Santarém is therefore essential to identify structural inequalities, recognize patterns of vulnerability and inform more equiTable public policies. An integrated analysis of these factors expands the understanding of how geographic, socioeconomic, cultural and organizational barriers shape the use of health services and their resolvability. Additionally, it highlights the urgency of strategies that strengthen Primary Health Care (PHC), expand service coverage and promote the right to health in its fullness, considering the specificities of traditional populations in the Amazon.

Methods

This study is characterized as an epidemiological, cross-sectional investigation with a quantitative approach, conducted to analyze geographic, organizational, and care-related accessibility to health services among quilombola communities in the municipality of Santarém, Pará, as well as to identify the distribution and patterns of self-reported diseases and factors associated with multimorbidity. The research was carried out in nine communities of the Lower Amazon region, Arapemã, Saracura, Pérola do

Maicá, Bom Jardim, João Pereira, Murumurutuba, Murumuru, Tiningu, and Ituqui, which present territorial heterogeneity marked by differences in distance from the municipal center, distinct environmental conditions, and modes of transportation influenced by river seasonality. These regional variations reflect diverse possibilities for accessing health services, justifying the choice of territory as the central axis of the analysis.

The study population consisted of 518 residents aged 18 years or older, living in the selected communities, and interviewed through a household survey. Data collection was conducted by trained researchers, ensuring greater accuracy of responses and adequate territorial understanding. The questionnaire included sociodemographic information, identification of up to 23 self-reported diseases, history of health service utilization, care resolvability, and characteristics related to mobility, transportation, and access difficulties. Specific variables such as “care in the last year,” “location of care,” “problem resolution,” and “health conditions” were used as key indicators for analyzing access and service performance.

After data collection, variables were standardized and statistically processed. Self-reported diseases were separated and converted into a binary matrix, allowing the calculation of prevalence by community and the identification of individuals with multimorbidity, defined as the presence of two or more simultaneous diseases. Geographic coordinates of communities and health services were obtained through latitude and longitude, enabling the construction of georeferenced maps representing territorial distribution and distance gradients. Mapping was conducted in Google Earth Pro, with later refinement in Google Maps, allowing integrated visualization of communities, health units, and relevant geographic routes. Geographic accessibility was classified into three levels (high, medium, and low), based on linear distance from each community to the urban area of Santarém.

Data analysis included the construction of health service utilization indicators, such as the proportion of care received in the last year, proportion of care performed outside the community, care resolvability, predominant educational level, and average income. These indicators were integrated into comparative analyses between communities, characterizing territorial and organizational disparities. Statistical tests included chi-square, ANOVA, and Spearman correlation to explore associations between variables, as well as a disease prevalence heatmap produced in Python using the Seaborn library.

To complement the territorial analysis, a Composite Access Index (CAI) was developed, integrating geographic dimensions, service-use indicators, and epidemiological elements to classify each community's access risk. The index was standardized on a 0-1 scale, with higher values representing poorer accessibility. Additionally, a Random Forest predictive model was applied to identify which conditions had the greatest importance in predicting multimorbidity among participants. The variables with the largest weight included highly prevalent chronic conditions such as hypertension, diabetes, and musculoskeletal diseases.

The study followed the ethical standards established by Resolution CNS No. 466/2012 and complied with the principles of the Declaration of Helsinki. Participant recruitment occurred between September 1, 2024, and October 30, 2025. All individuals invited to participate received detailed information about the study objectives and procedures, and written informed consent was obtained prior to data collection. Signed consent forms were securely stored as part of the study documentation, ensuring verification of voluntary participation. The research protocol was approved by the Research Ethics Committee of the Universidade do Estado do Pará (UEPA), under opinion No. 4.915.684, guaranteeing confidentiality, autonomy, and the protection of participants' rights throughout all stages of the research.

Results

Data from 518 residents belonging to the Quilombola communities of Santarém-PA were analyzed, focusing on the assessment of territorial and organizational accessibility to health services and the identification of epidemiological patterns associated with the risk of multimorbidity. The results are presented regarding the geographic classification of accessibility, indicators of service use, resolvability, integrated territorial risk, and predictive modeling.

Geographic classification of accessibility to the health system

Table 1 presents the criteria adopted for stratifying geographic accessibility based on the linear distance between the communities and the urban area of Santarém. This classification defines three levels: high, medium, and low accessibility, based on distances traveled, estimated travel time, and epidemiological implications.

Communities located up to 5 km from the urban zone were classified with high accessibility, suggesting a lower risk of physical barriers to regular access to health services. Distances greater than 5 km and up to 25 km comprised the medium accessibility category, indicating dependence on transportation and greater susceptibility to delays.

Communities situated more than 25 km away make up the low accessibility category, where there is a marked probability of care discontinuity, especially during periods of river seasonality.

Table 1. Criteria for classifying geographic accessibility based on distance and travel time

Classification	Distance (km)	Estimated Travel Time	Epidemiological Interpretation
High Accessibility	0–5 km	≤ 30 minutes	Favorable conditions for regular access; lower risk of territorial barriers.
Medium Accessibility	>5–25 km	30–90 minutes	Moderate access, subject to logistical limitations, dependent on available transport.
Low Accessibility	> 25 km	> 90 minutes	Difficult access; higher risk of delays, abandonment, and care discontinuity.

Accessibility to Primary Health Care (PHC)

Table 2 synthesizes accessibility to PHC services, considering distance, the presence of Basic Health Units (UBS) in the territory, and qualitative barrier analysis. It is noted that: Pérola do Maicá, located within the high accessibility radius, has greater ease of travel and better care continuity. Arapemã and Saracura, despite their proximity (6.5 and 9.9 km, respectively, in a straight line), are accessed by river transport, which is not regular and does not follow a straight line due to necessary detours for better navigability. Bom Jardim, João Pereira, and Murumurutuba show intermediate accessibility, marked by dependence on river or land transport, with direct impacts on response time and care regularity.

Murumuru and Ituqui represent scenarios of low accessibility, with distances exceeding 25 km and strong vulnerability associated with transport conditions. Tiningu, despite being classified in the Table as High due to having its own UBS, is located 40 km away, which characterizes low geographic accessibility, although with partial mitigation at the primary level, indicating that the presence of a UBS in the community does not eliminate territorial barriers, especially when there is great distance from diagnostic support services and medical referrals.

Table 2. Accessibility to Primary Health Care (PHC) services in the municipality of Santarém-PA, Lower Amazon region.

Community	Distance from Urban Area (km)	Classification	Available PHC Service	Accessibility Analysis
Pérola do Maicá	1.5	High	UBS Maicá	Best access condition; proximity favors care continuity.
Arapemã	6.5	Medium	UBS Tapará	Dependent on river travel.
Saracura	9.9	Medium	UBS Tapará	Dependent on river travel.
Bom Jardim	16.0	Medium	UBS Jacamim	Limited access; dependence on river/road transport.
João Pereira	20.0	Medium	UBS Jacamim	Limited access; dependence on river/road transport.
Murumurutuba	23.5	Medium	UBS Tiningú	Upper limit of medium accessibility; significantly longer travel.
Murumuru	25.0	Low	UBS Tiningú	Difficult access, especially during flood/dry seasons.
Tiningu	40.0	High*	UBS Tiningú (own)	Has its own UBS (but geographically distant).
Ituqui	30.0	Low	UBS Tapará Miri (region)	Difficult access; high territorial vulnerability.

Accessibility to medium complexity services

As shown in Table 3, most medium complexity services, such as the Elderly CRS, Child CRS, Women's CRS, and the "Melhor em Casa" Program, are concentrated in the urban perimeter of Santarém. Thus: Communities with high geographic accessibility

(Pérola do Maicá) have a structural advantage in accessing these services. Communities with medium and low accessibility face significant logistical barriers, such as long travel times, dependence on boats, and indirect costs, which can compromise the monitoring of chronic diseases and therapeutic continuity. This pattern reflects the urban centralization of specialized care provision and confirms inequalities in longitudinal care.

Table 3. Accessibility to medium complexity services in the municipality of Santarém-PA, Lower Amazon region.

Service	Communities with High Accessibility	Communities with Medium/Low Accessibility
Elderly CRS	Pérola do Maicá	Arapemã, Saracura, Bom Jardim, João Pereira, Murumurutuba, Murumuru, Tiningu, Ituqui
Child CRS	Pérola do Maicá	Arapemã, Saracura, Bom Jardim, João Pereira, Murumurutuba, Murumuru, Tiningu, Ituqui
Women's CRS	Pérola do Maicá	Arapemã, Saracura, Bom Jardim, João Pereira, Murumurutuba, Murumuru, Tiningu, Ituqui
Melhor em Casa Program	Pérola do Maicá	Arapemã, Saracura, Bom Jardim, João Pereira, Murumurutuba, Murumuru, Tiningu, Ituqui

Access to high complexity and emergency services

Table 4 highlights that, although services such as SAMU 192 (Mobile Emergency Service) are available to all communities, this is only at the level of activation. Structural barriers remain, especially related to response time in critical situations. For distant communities, the estimated time exceeds 60 minutes and can be worsened by river seasonality. In the field of mental health and infectious diseases, services such as CAPS AD III, CAPSi, and CTA/SAE have viable accessibility only for communities close to the urban area. Communities located more than 20 km away face substantial difficulties, with a direct impact on prevention, diagnosis, and timely treatment.

Table 4. Access to high complexity and emergency services in the municipality of Santarém-PA, Lower Amazon region.

Service	Type	Communities with Viable Access	Main Identified Barriers
SAMU 192	Emergency	All communities (via telephone)	Response time >60 min for distances >25 km; difficulty locating; river seasonality.
CEREST	Occupational Health	Pérola do Maicá	Distance and transport compromise access for more remote communities.
CAPS AD III	Mental Health	Pérola do Maicá	Access unviable for distant communities without regular transport.
CAPSi	Child Mental Health	Pérola do Maicá	Need for an accompanying adult limits adherence; prolonged travel.
CTA/SAE	STI/AIDS and Viral Hepatitis	Pérola do Maicá	Intense geographic barrier for communities >20 km; risk of late diagnosis.

Integrated accessibility classification

Table 5 presents the synthesis of accessibility classification considering PHC, medium, and high complexity. The communities were then categorized into four risk levels: Low risk: Pérola do Maicá, which has better infrastructure, shorter distance, and greater resolvability. Moderate risk: Arapemã, Saracura, Bom Jardim, and João Pereira, which depend on intermediate travel. Moderate/High risk: Murumurutuba, due to distance and transport fragility. High risk: Murumuru and Tiningu, with geographic barriers and dependence on external services. Very high risk: Ituqui, which concentrates the highest vulnerabilities. Communities with a distance greater than 25 km show high or very high risk, regardless of the presence of a UBS. This pattern confirms the influence of territorial inequalities on Quilombola health vulnerability.

Table 5. Territorial accessibility and integrated risk of the quilombola communities of Santarém-PA

Community	Distance from	PHC	Medium Complexity	High Complexity	Integrated Territorial Risk

	Urban Area (km)				
Pérola do Maicá	1.5	High	High	High	Low
Arapemã	6.5	High	Medium	Medium	Moderate
Saracura	9.9	High	Medium	Medium	Moderate
Bom Jardim	16	Medium	Low	Low	Moderate
João Pereira	20	Medium	Low	Low	Moderate
Murumurutuba	23.5	Medium	Low	Low	Moderate/High
Murumuru	25	Low	Low	Low	High
Tiningu	40	High*	Low	Low	High
Ituqui	30	Low	Low	Low	Very High

Figure 1 presents the spatial distribution, evidencing an unequal territorial pattern of accessibility to health services. A high concentration of health facilities is observed in the urban and peri-urban area of Santarém, especially near the Pérola do Maicá community, which is located practically within the urban fabric and therefore presents high geographic accessibility. This proximity results in greater service availability, shorter travel time, and greater care resolvability.

On the other hand, communities situated on the right bank of the Amazon River, such as Arapemã and Saracura, although geographically more distant from the urban area and accessed by intermittent river transport, present an intermediate pattern of connectivity and tend to access urban services with lower time cost. In contrast, Bom Jardim, João Pereira, Murumurutuba, and Murumuru are located in areas with lower service density and greater dependence on land and river routes, often influenced by flood and dry seasons. This spatial distribution reinforces the classification observed in the indicators: these communities present medium to low accessibility, which is associated with greater territorial vulnerability.

The Tiningu community, located approximately 40 km from the urban center, is the most evident example of geographic isolation. Despite having its own UBS, its distance from medium and high complexity services represents a significant barrier. The spatial pattern of Tiningu reflects high geographic vulnerability, which contributes to a

higher risk of care discontinuity and fewer opportunities to use specialized services (Figure 1).

Figure 1. Geographic distribution of Quilombola communities and health services in the municipality of Santarém-PA, Lower Amazon region.

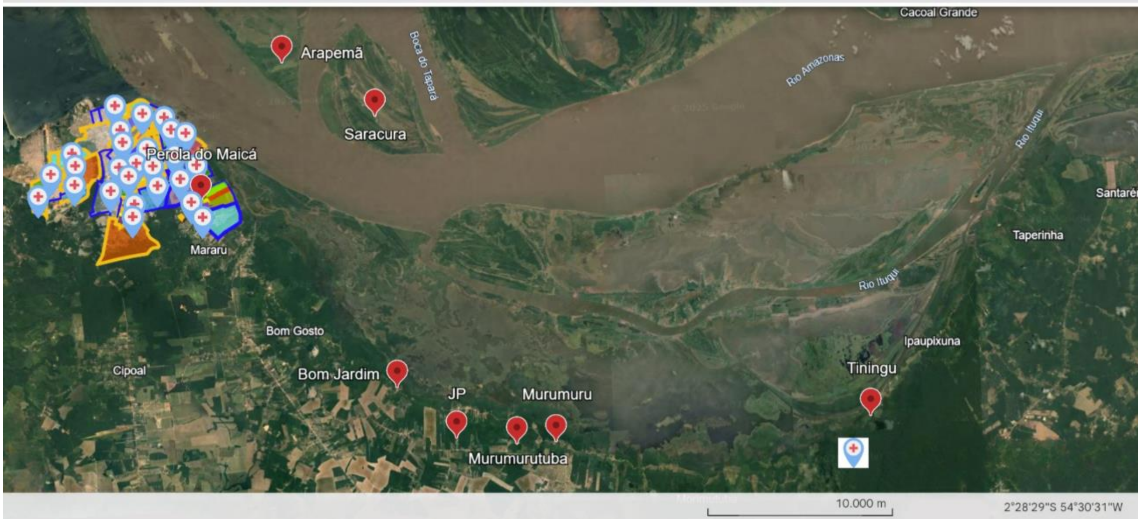

Indicators of Health Service Use

Table 6 presents data on service utilization, location of care, and resolvability. The results show that: The highest rates of utilization in the last year occurred in Pérola do Maicá (95.0%), Tiningu (85.7%), and João Pereira (82.6%). The percentage of care received outside the community was high in all locations (70–95%), highlighting the centralization of services in the urban area. Resolvability showed a high proportion of completely resolved problems in some communities, such as Arapemã (100%), but was low in others, such as Pérola do Maicá (57.1%), suggesting that greater utilization does not necessarily translate into higher quality of care.

Table 6 – Health service access and utilization indicators in the municipality of Santarém-PA, Lower Amazon region.

Community	Distance (km)	Care Last Year	Outside Community	Problem Resolved	Predominant Education Level	Average Income
Arapemã	6.5	42.9%	66.7%	100.0%	Elementary	3.25
Bom Jardim	16.0	62.5%	75.0%	83.3%	Elementary	2.83
João Pereira	20.0	82.6%	83.7%	85.7%	Elementary	2.96

Murumuru	25.0	81.8%	70.7%	78.6%	Elementary	3.33
Murumurutuba	23.5	64.3%	78.6%	80.0%	Elementary	3.75
Pérola do Maicá	1.5	95.0%	95.2%	57.1%	High School	3.33
Saracura	9.9	72.0%	76.9%	70.0%	High School	2.50
Tiningu	40.0	85.7%	72.7%	72.7%	Elementary	3.80
Ituqui	30.0	N/A	N/A	N/A	Elementary	2.70

Table 7 shows that 72.5% of participants reported their problem was completely resolved, while 15.4% reported partial resolution. Only 4.0% reported that their demand was not met, and 8.1% did not know or did not respond. These results show a relatively satisfactory care performance, but still with heterogeneity among communities and a need to improve the quality of care provided.

Table 7 – Classification of health care resolvability in the municipality of Santarém-PA, Lower Amazon region.

Status	n	%
Problem completely resolved	198	72.5%
Problem partially resolved	42	15.4%
Problem not resolved	11	4.0%
Not informed / indeterminate	22	8.1%
Total	273	100.0%

Composite Access Index (CAI)

Table 8 presents the CAI, constructed from the integration of distance, disease burden, and social indicators. The results show that: Ituqui (0.550), Tiningu (0.480), and Murumurutuba (0.331) presented the highest values, reflecting greater territorial vulnerability. Pérola do Maicá (0.076) presented the best index, compatible with its proximity to the urban zone. The CAI reinforces structural inequalities and supports the prioritization of differentiated strategies among territories.

Table 8 – Classification of Quilombola communities according to the Composite Access Index (CAI)

Community	Average CAI
Ituqui	0.550
Tiningu	0.480
Murumurutuba	0.331
Murumuru	0.324
João Pereira	0.275
Bom Jardim	0.253
Saracura	0.191
Arapemã	0.139
Pérola do Maicá	0.076

The Random Forest modeling (Table 9) had excellent performance, with overall accuracy of 93.6%, allowing for the identification of determining factors for the occurrence of multimorbidity. The variables with the greatest importance were: Hypertension, Diabetes, Spinal disease, Arthritis/Rheumatism, Heart disease. These results corroborate the literature on chronic conditions as the central axis of the epidemiological profile of vulnerable populations.

Table 9 – Variable importance in the multimorbidity predictive model (Random Forest) in the municipality of Santarém-PA, Lower Amazon region.

Variable	Importance
Hypertension	0.192
Diabetes	0.111
Spinal Disease	0.088
Arthritis/Rheumatism	0.072
Heart Disease	0.067

The heatmap, Figure 2, highlights distinct patterns in the distribution of chronic and infectious diseases among the Quilombola communities. Greater density is observed in João Pereira and Murumurutuba, with intermediate prevalences of diabetes, spinal

diseases, and arthritis/rheumatism, suggesting a more homogeneously distributed chronic morbidity profile in these communities. The presence of lighter shades in Tiningu for conditions like "Not applicable" indicates a high proportion of unreported diagnoses, which may point to low detection, underdiagnosis, or lower frequency of visits to services providing formal diagnosis. This pattern is consistent with the high geographic distance and the territorial risk classification attributed to this community.

Communities near the urban center, such as Pérola do Maicá, Arapemã, and Saracura, show more dispersed patterns, with low to moderate prevalences across various conditions, indicating more regular access and more distributed diagnosis. In Saracura, greater intensity in conditions like spinal disease and asthma stands out, suggesting a profile potentially associated with occupational exposure, physical strain, and environmental conditions.

Infectious diseases, such as malaria, dengue, and hepatitis, appear sporadically and with lower intensity, reflecting reduced prevalences in the communities, but with variations that suggest differentiated ecological exposure, especially in Murumuru and Murumurutuba, where there are moderate records of endemic Amazonian diseases.

Figure 2. Heatmap of disease prevalence by Quilombola community in the municipality of Santarém-PA.

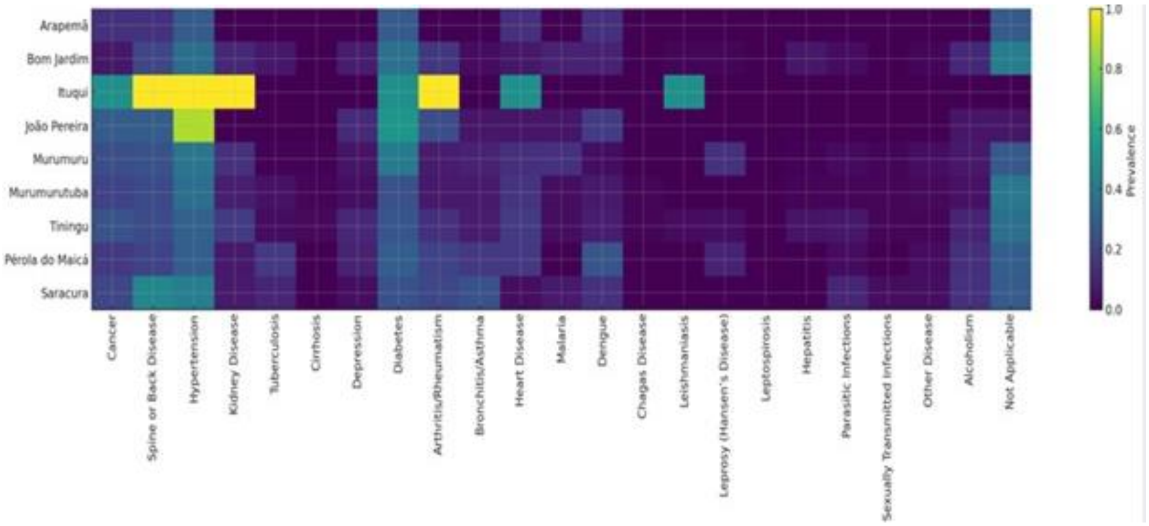

Discussion

The findings reveal a scenario that aligns closely with what various authors have described regarding the structural inequalities that shape the lives of quilombola populations. Understanding these inequalities becomes stronger when analyzed through

theoretical models addressing the social determination of health, particularly those that describe how environmental, socioeconomic, and political conditions shape access to services and influence patterns of illness within communities. In this regard, the framework proposed by Dahlgren and Whitehead stands out by illustrating successive layers of determination from individual factors to broader social structures, thus helping interpret the heterogeneity observed among the communities analyzed [10].

The set of geographic, economic, and organizational barriers identified in the quilombola territory also echoes the perspective of the National Commission on Social Determinants of Health, which considers access as a phenomenon conditioned by multiple interdependent elements rather than by the mere physical presence of services [11]. Analyzing access from this standpoint reveals that communities farther from urban centers tend to accumulate obstacles that extend beyond the territorial dimension, including indirect costs, dependence on river transportation, logistical difficulties, and reduced availability of specialized care.

In light of Michael Marmot's theory of health inequities, these findings reinforce the understanding that territorial differences are not random but reflect historically produced social structures that result in unequal opportunities and living conditions [2,12]. The unequal distribution of health services, especially those of medium and high complexity contributes to the intensification of a cycle in which the most vulnerable populations face greater difficulty accessing care, reduced care continuity, and, in some cases, an increased risk of adverse outcomes. This interpretation positions geography as an inherent component of inequity rather than a mere environmental factor.

The literature on quilombola health demonstrates that vulnerabilities manifest not only through territorial restrictions but also through the organization of services and the degree to which institutional systems recognize their needs [13]. Studies across different regions highlight that geographic distance, though important, represents only one facet of the issue, as it interacts with income, education, transportation availability, environmental conditions, and cultural barriers to shape real experiences of accessing the SUS [14,15]. This pattern is confirmed in Santarém, where more isolated communities accumulate multiple layers of disadvantage.

Additionally, the World Health Organization recognizes that unfavorable socioeconomic conditions are associated with higher disease burden and reduced ability to seek timely care [16]. Among quilombola populations, such factors include limited access to income and education as well as historically rooted racial vulnerabilities, as

discussed by Bailey et al. in their analysis of structural racism and health [17]. This theoretical framework supports the interpretation of the multimorbidity patterns observed, particularly in communities with limited accessibility.

Research on quilombola health also indicates that traditional care practices, communication challenges with health professionals, and previous experiences of discrimination can influence service use and trust in the health system [18,19]. In Santarém, findings related to resolvability show that higher utilization does not necessarily translate into improved care experiences, suggesting possible weaknesses in care continuity, effectiveness, and patient reception.

The role of nursing in these territories also deserves emphasis. Studies indicate that professionals frequently face resource shortages, work overload, and logistical limitations, especially in rural and riverine areas [20–22]. Such conditions affect both the quality of care and the ability to perform surveillance, prevent diseases, and monitor chronic conditions. These limitations tend to be more pronounced in remote communities.

Another important aspect concerns emergency services in Amazonian territories. Although national policy expanded mobile emergency coverage through river ambulances (Ambulanchas), studies indicate persistent difficulties related to vessel maintenance, unsTable communication systems, and long response times [23,24]. Our findings align with these challenges, particularly in more isolated areas such as Tiningu and Ituqui, where residents often rely on private transport in critical situations.

From a public policy perspective, authors such as Freitas, Panno, and Almeida emphasize that the historical trajectory of quilombola communities in the Amazon has been marked by the formation of resistance territories that continue to face structural barriers to fully exercising constitutional rights, including health [25–27]. These challenges become evident when observing the distribution of health units, the centralization of specialized services in urban areas, and the need for long-distance travel for examinations and consultations.

Furthermore, health promotion as discussed by Durand and Heideman requires acknowledging sociocultural and environmental dimensions that shape ways of life and conditions of illness [28]. In quilombola communities, daily practices, forms of community organization, and support networks are essential to understanding both care-seeking behavior and decisions related to self-care.

When examining the patterns of multimorbidity and service utilization identified, it becomes evident that social, racial, and territorial inequalities intersect,

disproportionately affecting residents' health, as demonstrated in recent studies of quilombola regions [29–31]. The literature reinforces that Black and traditional populations face a higher burden of chronic diseases, often exacerbated by barriers to access, delayed diagnoses, and inconsistent follow-up.

The difficulty in accessing health information, highlighted by Norman and Skinner in their concept of digital health literacy, must also be considered [32]. In communities with limited connectivity, obtaining reliable information on prevention, disease management, and navigating the health system is challenging, directly influencing service use and self-care capacity.

Finally, recent studies on therapeutic itineraries in the Amazon show that the path to care is shaped by complex decisions involving time, cost, climate, transportation availability, and expectations regarding care [33]. In the communities analyzed, these factors resonate strongly with findings related to problem resolution, place of first care, and reliance on services outside the community.

Taken together, the findings suggest that health access in quilombola communities is a multifaceted phenomenon shaped by overlapping structural, historical, social, cultural, and organizational conditions. Understanding how these layers interact is essential for guiding territorially sensitive public policies capable of promoting greater equity in health.

Conclusion

This study demonstrates that health access in quilombola communities of the Amazon is shaped by a complex interplay of territorial, social and organizational determinants that extend far beyond the mere availability of services. Rather than a linear relationship between distance and care, the findings reveal the influence of historically produced inequities, transportation fragilities, and structural constraints that limit the capacity of these communities to obtain timely diagnosis, continuous follow-up and effective management of chronic conditions. These dynamics underscore the urgency of policies that move beyond standardized solutions and instead incorporate territorial nuances, community-specific vulnerabilities and culturally grounded approaches to care.

However, the findings must be interpreted in light of important limitations. The study relies on self-reported morbidity, which may underestimate disease prevalence due to memory bias, lack of previous diagnoses and limited access to diagnostic services, particularly in more isolated territories, where underdiagnosis is structurally embedded. The cross-sectional design restricts causal inference, preventing the establishment of

temporal relationships between accessibility, service use and multimorbidity. Geographic accessibility was evaluated using linear distances, which do not fully capture the effects of river dynamics, climatic variability, transportation instability or seasonal navigability, factors that strongly influence real travel times in the Amazon. Additionally, the Random Forest model identifies associations but does not account for contextual factors such as cultural practices, traditional medicine or trust in the health system, which may shape health-seeking behaviors in ways that quantitative models cannot capture.

Despite these limitations, the study offers a territorialized perspective essential for guiding more equiTable public health strategies. Strengthening primary care, improving transportation and communication infrastructures and incorporating community participation in planning are critical steps toward mitigating systemic vulnerabilities. Future research should integrate longitudinal data, qualitative insights and geospatial models that better reflect riverine mobility to deepen the understanding of health inequities in traditional Amazonian territories.

Abbreviations:

ANOVA – Analysis of Variance
CAAE – Certificate of Ethical Approval Submission
CAI – Composite Access Index
CAPS AD III – Psychosocial Care Center for Alcohol and Drugs
CAPSi – Child Psychosocial Care Center
CEP – Research Ethics Committee
CEREST – Occupational Health Reference Center
CRS – Specialized Reference Center (e.g., Elderly, Women, Child)
CTA/SAE – Counseling and Testing Center / Specialized Assistance Service
PHC – Primary Health Care
SDH – Social Determinants of Health
SAMU 192 – Mobile Emergency Care Service
SUS – Unified Health System
UBS – Basic Health Unit
WHO – World Health Organization

Ethics approval and consent to participate: The study was approved, under approval number 4.915.684 and CAAE 46491621.1.0000.5168, by the Research Ethics Committee (CEP) of the State University of Pará, located at Rua Plácido de Castro, 1399,

Bairro Aparecida, Santarém, PA, CEP: 68038610, e-mail: ceptapajos@uepa.br, informed consent was obtained from all participants and all methods were performed in accordance with current guidelines and standards. Data collection was conducted from April 10, 2024, to September 17, 2025. The study adhered to the ethical principles outlined in the Declaration of Helsinki, ensuring respect for human dignity, autonomy, and the protection of participants' rights throughout all stages of the research.

Consent for publication: Not applicable.

Availability of data and materials: The data analyzed in this study are not publicly available due to privacy and ethical restrictions. The dataset used for this research contains sensitive patient in-formation and is subject to confidentiality agreements. Therefore, the data cannot be shared openly. However, researchers interested in accessing the data can contact the corresponding author and request access, subject to the approval of the ethics committee and compliance with relevant data protection regulations.

Competing Interests: The authors declare no conflict of interest.

Funding: This research received no external funding.

Authors' contributions: Leanna Silva Aquino participated in the conception and design of the study, article writing, data analysis and interpretation, and critical review of the article. Ellen Mara Fernandes da Silva participated in the conception and design of the study, article writing, data analysis and interpretation, and critical review of the article. Victoria Valentim Aguiar participated in the conception and design of the study, article writing, data analysis and interpretation, and critical review of the article. Cesar Ferreira Fernandes Filho participated in the conception and design of the study, article writing, data analysis and interpretation, and critical review of the article. Sheyla Mara Silva de Oliveira participated in the conception and design of the study, article writing, data analysis and interpretation, and critical review of the article. Tatiane Costa Quaresma participated in the conception and design of the study, article writing, data analysis and interpretation, and critical review of the article. Valney Mara Gomes Conde participated in the conception and design of the study, article writing, data analysis and interpretation, and critical review of the article. Nádia Vicência do Nascimento Martins participated in the conception and design of the study, article writing, data analysis and interpretation, and critical review of the article. Marcos Manoel Honorato participated in the conception and design of the study, article writing, data analysis and interpretation, and critical review of the article. Veridiana Barreto do Nascimento participated in the conception and

design of the study, article writing, data analysis and interpretation, and critical review of the article. Guilherme Augusto Barros Conde participated in the conception and design of the study, article writing, data analysis and interpretation, and critical review of the article. Franciane de Paula Fernandes participated in the conception and design of the study, article writing, data analysis and interpretation, and critical review of the article. Lívia de Aguiar Valentim participated in the conception and design of the study, article writing, data analysis and interpretation, and critical review of the article.

Acknowledgements: We would like to thank the partnership with the project "Connecting Knowledge in the Amazon: Integrating Quilombola Communities and Public Schools with Computing and Artificial Intelligence", approved through a public call by the National Council for Scientific and Technological Development (CNPq) and carried out by the Federal University of Western Pará (UFOPA) and Project "Weaving Networks of Health and Knowledge: Innovation and Tradition in the Amazon", approved through a public call by the National Council for Scientific and Technological Development (CNPq) and carried out by the University of State Pará (UEPA).

References

1. World Health Organization. Health inequities and their causes. Geneva: WHO; 2018.
2. Marmot M. Social determinants of health inequalities. Lancet. 2005;365(9464):1099–104.
3. Fiorati RC, Arcêncio RA, Souza LB. Social inequities and access to health: reflections based on the principle of integrality. Rev Atenção Prim Saúde. 2016.
4. Commission on Social Determinants of Health (CNDSS). Final Report — The social causes of health inequities in Brazil. Brasília: Ministério da Saúde; 2008. Available from: https://bvsms.saude.gov.br/bvs/publicacoes/causas_sociais_iniquidades.pdf
5. Torres GMC, et al. Access and integrality in the health care of quilombola women: challenges to equity. Research, Society and Development. 2022;11(9):e57011932158. doi: 10.33448/rsd-v11i9.32158
6. Amador EO, et al. Social determinants of health as an impact factor in health care for quilombola populations: a systematic review. Rev Eletrônica Acervo Saúde. 2024;24(4):e14922. Available from: https://acervomais.com.br/index.php/saude/article/view/14922
7. Cavalcante IMS, Silva HP. Public policies and access to health services in quilombos of the Amazon. In: Quilombolas: political, legal and inclusive policies arising from

Decree 4887/2003 and ADI 3239. Rio de Janeiro: TRF-2; 2019. p. 473–98. Available from: https://www.researchgate.net/publication/335326369

8. Pereira RDN, Mussi RF de F. Access and use of health services among Black quilombola populations: a bibliographic analysis. ODEERE. 2020;5(10):280–303. Available from: https://periodicos2.uesb.br/index.php/odeere/article/view/6938
9. Lança EFC, Sobral LL, Vieira RCA, Nogueira LS, Sousa RMC. River emergency service: characteristics and factors related to clinical evolution of assisted patients. Cad Saúde Pública. 2021;37(8):e00281120. Available from: https://www.scielosp.org/article/csp/2021.v37n8/e00281120/
10. Commission on Social Determinants of Health (CSDH). Closing the gap in a generation: health equity through action on the social determinants of health. Geneva: WHO; 2008.
11. Dahlgren G, Whitehead M. Policies and strategies to promote social equity in health. Stockholm: Institute for Futures Studies; 1991.
12. Santos L, Santos RC, Ferreira JL, Rico AM, Almeida TS. Approaches and perceptions of health inequities in medical education: a scoping review. Rev Bras Educ Med. 2025;49(2):e079. doi: 10.1590/1981-5271v49.2-2023-0259
13. Torres GMC, et al. Access and integrality in health care of quilombola women: challenges to equity and health rights. Research, Society and Development. 2022;11(9):e57011932158. doi: 10.33448/rsd-v11i9.32158
14. Oliveira TS, Pereira AMM. Expressions of inequalities in access to health services in Latin America: a scoping review. Ciênc Saúde Coletiva. 2024;29(7):e04932024. doi: 10.1590/1413-81232024297.04932024
15. Sousa RF, Rodrigues ILA, Pereira AA, Nogueira LMV, Andrade EGR, Pinheiro AKC. Health conditions and use of services from the perspective of quilombola people. Esc Anna Nery. 2023;27:e20220164. doi: 10.1590/2177-9465-EAN-2022-0164pt
16. Kirkbride JB, Anglin DM, Colman I, Dykxhoorn J, Jones PB, et al. Social determinants of mental health and disorder: evidence, prevention and recommendations. World Psychiatry. 2024;23(1):58–90. doi: 10.1002/wps.21160
17. Bailey ZD, et al. Structural racism and health inequities in the USA. Lancet. 2017;389:1453–63.
18. Silva LJW, Prudêncio LS, Santana PP, Alves VH, Calandrini TSSS, Cavalcante SVPM, Mata NDS. Therapeutic itineraries and health care networks of high-risk pregnant women in the Amazon. Rev Enferm Atual In Derme. 2025;99(Suppl

1):e025088. Available from: https://mail.revistaenfermagematual.com.br/index.php/revista/article/view/2414

19. Souza BO. Aquilombar-se: historical, identity and political panorama of the Brazilian Quilombola Movement. Brasília: Universidade de Brasília; 2008. Available from: http://icts.unb.br/jspui/handle/10482/2130
20. Cardoso CS, de Melo LO, Freitas DA. Health conditions in quilombola communities. Rev Enferm UFPE on line. 2018;12(4):1037–45. Available from: https://periodicos.ufpe.br/revistas/revistaenfermagem/article/view/110258
21. Braghetto GT, Sousa LA, Beretta D, Vendramini SHF. Difficulties and facilitators perceived by Family Health Strategy nurses in their work process. Cad Saúde Colet. 2019;27(4):420–6. doi: 10.1590/1414-462X201900040100
22. Lima EA, Santana JE, Matias RA, Araujo TA, Barbosa RS. Challenges of nursing in the Unified Health System. Rev Interdiscip Encontro das Ciências – RIEC. 2021;4(3):409–14.
23. Silva MCN, Machado MH. Health system and work: challenges for nursing in Brazil. Ciênc Saúde Coletiva. 2020;25(1):7–13. doi: 10.1590/1413-81232020251.27572019
24. Ferreira D, Arcuri R, Bulhões B, Bellas HC, Masson L, Vidal MC, Jatobá A, Carvalho PVR. Challenges in maintaining the Mobile River Emergency Service in Alto Solimões. Rev Human Inov. 2020;7(16):494–505.
25. Freitas DA, Caballero AD, Marques AS, Hernández CIV, Antunes LNO. Health and quilombola communities: a literature review. Rev CEFAC. 2011;13(5):937–43. doi: 10.1590/S1516-18462011005000033
26. Panno M. History of the occupation of the Amazon. In: Tom da Amazônia—Teacher's guide. Rio de Janeiro: Fundação Roberto Marinho; 2005. Available from: https://www.yumpu.com/pt/document/view/13654946/
27. Almeida RR. Slavery, resistance, and the formation of quilombos in the Amazon: the case of Jacarequara. In: XVII National Symposium of History. Natal, Brazil; 2013. p. 1–17.
28. Durand MK, Heideman ITS. Social determinants of a quilombola community and its interface with health promotion. Rev Esc Enferm USP. 2019;53:e03451.
29. Cavalcante IMS, Silva HP. Public policies and access to health services in Amazon quilombos. In: Quilombolas: political, legal and inclusive policies. Rio de Janeiro: TRF-2; 2019. p. 473–98.

30. Marques YFO, Silva YJC, Karnikowski IGO, et al. Scoping review on quilombola communities in Brazil's Central-West region: health and culture in focus. Rev Diálogos Gerontol. 2023.
31. Costa AK, Bertoldi AD, Fontanella AT, Ramos LR, Arrais PSD, Luiza VL, et al. Is there socioeconomic inequality in multimorbidity among Brazilian adults? Rev Saude Publica. 2020;54:138. doi: 10.11606/s1518-8787.2020054002569
32. Norman CD, Skinner HA. eHealth literacy: essential skills for consumer health. J Med Internet Res. 2006;8(2):e9. doi: 10.2196/jmir.8.2.e9
33. Silva LJW, Prudêncio LS, Santana PP, Alves VH, Calandrini TSSS, Cavalcante SVPM, Mata NDS. Therapeutic itinerary and health care network of high-risk pregnant women in the Amazon. Rev Enferm Atual In Derme. 2025;99(Suppl 1):e025088. Available from: https://revistaenfermagematual.com.br/index.php/revista/article/view/2414
