# Supplementary material for: Geographical barriers and multimorbidity in quilombola territories of the amazon region
Source: PLoS One. 2026 Mar 27;21(3):e0344043. doi: 10.1371/journal.pone.0344043 (PMC13028379; doi:10.1371/journal.pone.0344043)
Supplement: Attachment — Submitted filename: Dear Editor and Reviewers plos one.pdf [file pone.0344043.s003.pdf]

Dear Editor and Reviewers,

We would like to thank the reviewers for their careful reading of our manuscript and for the constructive and insightful comments provided. We are particularly grateful for the positive assessment of the study's relevance, conceptual framework, and the effort involved in data collection in remote quilombola territories. We have carefully revised the manuscript to improve methodological clarity, analytical rigor, transparency, and consistency with the cross-sectional design. Below, we provide a point-by-point response detailing how each comment was addressed.

We sincerely thank Reviewer for the positive evaluation of the study and for recognizing its potential contribution to public policies aimed at quilombola communities. The encouraging feedback regarding the coherence between objectives and methodology is greatly appreciated and motivated us to further strengthen the manuscript.

Major Comments Reviewer 2

1. Causal language and cross-sectional design

Reviewer comment: The study objective implies a causal relationship between geographical barriers and multimorbidity. Given the cross-sectional design, the authors should clarify that the study evaluates associations rather than causal effects.

Response: We revised the language throughout the manuscript, including the Abstract, Results, Discussion, and Conclusions, to consistently emphasize associational relationships rather than causal effects. Specifically, causal expressions were replaced with terms such as "are associated with", "are related to", and "are linked to". In addition, we inserted an explicit statement in the Discussion clarifying that, due to the cross-sectional design, the findings should be interpreted as associations and that temporal or causal inferences cannot be established.

2. Age adjustment and standardization

Reviewer comment: Given the strong association between age and multimorbidity, age-adjusted or age-standardized analyses could strengthen comparisons across communities.

Response: We acknowledge the importance of age as a key determinant of multimorbidity. However, given the study's exploratory and territorial focus, as well as the relatively small number of communities, age-standardized analyses at the community level were not conducted. We explicitly addressed this limitation in the Discussion, noting that differences in age structure across communities may partially explain variations in multimorbidity prevalence. We also emphasized that future studies should incorporate age-standardized estimates or multivariable models to strengthen comparative analyses.

3. Participant selection and selection bias

Reviewer comment: The manuscript does not clearly describe how participants were selected within each community, nor does it report refusals or non-response rates.

Response: We substantially expanded the Methods section to clarify the sampling strategy. Participant selection is now explicitly described as non-probabilistic consecutive sampling, combining territorial and institutional recruitment approaches. Inclusion and exclusion criteria were clearly defined, and the recruitment contexts were detailed (health unit, community meetings, household visits, and itinerant health actions). We also reported that refusals and non-responses were infrequent and not concentrated in specific communities.

4. Inconsistency in data collection period

Reviewer comment: There is an inconsistency regarding the reported period of data collection.

Response: We carefully reviewed and harmonized all references to the data collection period across the manuscript. The Methods and Ethics sections now consistently report that data collection occurred between September 1, 2024, and October 30, 2025, ensuring internal consistency.

5. Definition of self-reported diseases

Reviewer comment: The manuscript does not clarify whether self-reported diseases refer to prior diagnoses, symptoms, or medication use.

Response: We revised the Methods section to clearly state that health conditions were assessed based on self-reported previous diagnoses communicated by health professionals (physicians or nurses). Symptoms in the absence of a prior diagnosis were not considered. We also explained that examples of medications and exams were used only to aid recall. In the Discussion, we expanded the interpretation to address the implications of self-reported morbidity and the potential for underdiagnosis in contexts with limited access to diagnostic services.

6. Composite Access Index (CAI): methodological transparency

Reviewer comment: Additional details on the CAI formulation, standardization, weighting, and rationale are needed.

Response: We substantially expanded the Methods section describing the Composite Access Index. The revised text now includes: the explicit mathematical formula; the min–max normalization procedure; the equal-weighting scheme; and the rationale for adopting equal weights to avoid arbitrary prioritization.

Additionally, we included an exploratory sensitivity analysis using alternative weighting schemes and reported that the relative ranking of communities remained stable. A supplementary table presenting raw and standardized values for CAI components was added to enhance transparency and reproducibility.

7. Difference between sample sizes (n = 273 vs. n = 518)

Reviewer comment: Clarification is needed regarding why the sample size in one table differs from the total study sample.

Response: We clarified throughout the Methods, Results, and table legends that some analyses were conducted using specific subgroups. In particular, the resolvability analysis was restricted to participants who reported having accessed health services in the previous 12 months (n = 273). Denominators (N) are now explicitly reported in the relevant tables and figure legends, and an explanatory note was added to Table 7 to avoid misinterpretation.

8. Random Forest analysis: methodological details

Reviewer comment: More information is needed on model validation, class balance, hyperparameter tuning, and performance metrics beyond accuracy.

Response: We expanded the Methods section to include a detailed description of the Random Forest implementation, including: training/testing split (70/30); class imbalance assessment and class-weight adjustment; hyperparameter tuning using grid search; handling of missing data through complete-case analysis; and performance evaluation using sensitivity, specificity, and AUC in addition to overall accuracy.

9. Interpretation of Random Forest variable importance

Reviewer comment: Variable importance measures should be interpreted as predictive relevance rather than causal or explanatory effects.

Response: We revised the Discussion to explicitly clarify that Random Forest variable importance reflects predictive relevance and not causal or etiological effects. We emphasized that the identified conditions should be interpreted as markers that co-occur with multimorbidity rather than independent risk factors, ensuring appropriate interpretation of the machine learning results.

Major Comments Reviewer 3

1. Sampling strategy and representativeness

Comment: The manuscript would benefit from a more detailed description of participant selection, eligibility, refusals, and non-responses, as well as a participant flowchart.

Response: We expanded the Methods section to provide a detailed description of the sampling strategy, clarifying that participant selection followed a non-probabilistic consecutive sampling approach combining territorial and institutional recruitment. Inclusion and exclusion criteria were explicitly defined. We also clarified the recruitment contexts, reported refusals and non-responses, and emphasized that losses were minimal and not concentrated in specific communities.

2. Inconsistencies in sample size and denominators

Comment: Some tables present denominators that differ from the total sample size without clear explanation.

Response: We clarified throughout the Methods, Results, and table footnotes that some analyses were conducted using specific subgroups, depending on data availability and analytical relevance. In particular, analyses related to health care resolvability were restricted to participants who reported health service use in the previous 12 months (n = 273). Denominators (N) are now explicitly reported and consistently described in all relevant tables and figure legends.

3. Definition and measurement of health conditions

Comment: The manuscript does not clearly specify whether health conditions were based on prior diagnoses, symptoms, or medication use.

Response: We revised the Methods section to clarify that health conditions were assessed through self-reported previous diagnoses communicated by health professionals. Symptoms without prior diagnosis were not considered. We also expanded the Discussion to address the implications of self-reported morbidity, particularly the potential for underdiagnosis in settings with limited access to diagnostic services.

4. Composite Access Index (CAI): transparency and reproducibility

Comment: Additional methodological details regarding the CAI are needed, including formula, normalization, weighting, and sensitivity analysis.

Response: We substantially expanded the description of the Composite Access Index in the Methods section. The mathematical formulation, min–max normalization procedure, equal-weighting scheme, and interpretation of index values were explicitly described. A supplementary table presenting raw and standardized component values was added. We also included an exploratory sensitivity analysis demonstrating the stability of community rankings under alternative weighting schemes, which is described in the Methods and referenced in the Results.

5. Measurement of geographic distance

Comment: The use of Euclidean distance may underestimate real travel distance or time in Amazonian contexts.

Response: We added a dedicated paragraph to the Discussion explicitly addressing this limitation. We justified the use of linear distance based on data availability, transparency, and comparability, while acknowledging its limitations in riverine and rural Amazonian settings. We also discussed how distance was integrated into the CAI alongside service-use and epidemiological indicators and highlighted future directions incorporating travel time or network-based measures.

6. Statistical analyses and hierarchical data structure

Comment: The assumption of independent observations may not fully hold due to clustering within communities.

Response: We acknowledge this limitation in the Discussion and clarified that the statistical analyses were primarily exploratory and descriptive. We added text noting that the hierarchical structure of the data may lead to underestimated uncertainty and that future studies should apply multilevel or mixed-effects models adjusting for individual-level confounders.

7. Random Forest analysis: methodological details and interpretation

Comment: More details are needed regarding model implementation, performance metrics, and interpretation of variable importance.

Response: We expanded the Methods section to describe the Random Forest implementation, including training–testing split, class imbalance handling, hyperparameter tuning, missing data treatment, and evaluation metrics beyond accuracy (sensitivity, specificity, and AUC). Additional performance metrics were included in supplementary material. We also revised the Discussion to clearly state that variable importance reflects predictive relevance rather than causal or explanatory effects.

8. Age structure and confounding factors

Comment: Comparisons between communities were not adjusted for age.

Response: We acknowledged this limitation in the Discussion, emphasizing that differences in age structure may partially explain observed variations in multimorbidity prevalence. We clarified that the analyses aimed to explore territorial patterns rather than estimate causal effects and noted that future studies should incorporate age-standardized estimates or multivariable adjustment.

9. Cross-sectional design and causal language

Comment: Some statements suggest causal relationships despite the cross-sectional design.

Response: We carefully revised the Abstract, Results, and Discussion to replace causal language with associational terminology throughout the manuscript. An explicit statement was added to the Discussion clarifying that findings represent associations rather than causal effects, ensuring alignment with the cross-sectional design.

10. Ethical and procedural clarifications

Comment: There are inconsistencies in reported data collection dates and insufficient detail regarding informed consent procedures.

Response: We harmonized all data collection dates across the manuscript. We also clarified the informed consent process, explaining that consent was obtained in written or verbally assisted form when necessary, with appropriate explanation and support to address potential literacy barriers.

Minor Comments

Figure and table legends: All legends were revised to include sample sizes, category definitions, and explanations of color scales for maps and heatmaps.

Results vs. Discussion: Interpretative statements were moved from the Results section to the Discussion to improve structural clarity.

Language and formatting: The manuscript underwent careful language revision to correct grammatical, formatting, and capitalization issues.

Missing data: We clarified the handling of missing data and "Not applicable" responses in the Methods section.

We believe that these revisions substantially improve the clarity, transparency, and methodological rigor of the manuscript while preserving its original objectives and contributions. We sincerely thank the reviewers for their thoughtful and constructive feedback, which greatly strengthened the quality of this work.

Sincerely,
Lívia de Aguiar Valentim
On behalf of all authors
